# Ammonia- and Nitrite-Oxidizing Bacteria are Dominant in Nitrification of Maize Rhizosphere Soil Following Combined Application of Biochar and Chemical Fertilizer

**DOI:** 10.3389/fmicb.2021.715070

**Published:** 2021-10-05

**Authors:** Ping Sun, Ziting Zhao, Pingshan Fan, Wei Chen, Yunze Ruan, Qing Wang

**Affiliations:** Hainan Key Laboratory for Sustainable Utilization of Tropical Bioresources, College of Tropical Crops, Hainan University, Haikou, China

**Keywords:** nitrification, AOA, AOB, Comammox *Nitrospira*, NOB, rhizosphere soil

## Abstract

Autotrophic nitrification is regulated by canonical ammonia-oxidizing archaea (AOA) and bacteria (AOB) and nitrite-oxidizing bacteria (NOB). To date, most studies have focused on the role of canonical ammonia oxidizers in nitrification while neglecting the NOB. In order to understand the impacts of combined biochar and chemical fertilizer addition on nitrification and associated nitrifiers in plant rhizosphere soil, we collected rhizosphere soil from a maize field under four different treatments: no fertilization (CK), biochar (B), chemical nitrogen (N) + phosphorus (P) + potassium (K) fertilizers (NPK), and biochar + NPK fertilizers (B + NPK). The potential nitrification rate (PNR), community abundances, and structures of AOA, AOB, complete ammonia-oxidizing bacteria (Comammox *Nitrospira* clade A), and *Nitrobacter*- and *Nitrospira*-like NOB were measured. Biochar and/or NPK additions increased soil pH and nutrient contents in rhizosphere soil. B, NPK, and B + NPK treatments significantly stimulated PNR and abundances of AOB, Comammox, and *Nitrobacter*- and *Nitrospira*-like NOB, with the highest values observed in the B + NPK treatment. Pearson correlation and random forest analyses predicted more importance of AOB, Comammox *Nitrospira* clade A, and *Nitrobacter*- and *Nitrospira*-like NOB abundances over AOA on PNR. Biochar and/or NPK additions strongly altered whole nitrifying community structures. Redundancy analysis (RDA) showed that nitrifying community structures were significantly affected by pH and nutrient contents. This research shows that combined application of biochar and NPK fertilizer has a positive effect on improving soil nitrification by affecting communities of AOB and NOB in rhizosphere soil. These new revelations, especially as they related to understudied NOB, can be used to increase efficiency of agricultural land and resource management.

## Introduction

To meet the food needs of a large population, excess chemicals are often applied in agricultural production (Liu et al., 2015). However, chemical fertilizer use efficiency in Chinese agricultural ecosystem is typically low (Zhang et al., 2015). At the same time, the overuse and low use efficiency of chemical fertilizers have led to serious environmental problems such as soil acidification (Guo et al., 2010), increased greenhouse gas emissions (Hu et al., 2015), and surface water and groundwater pollution (Sebilo et al., 2013). This pathway is closely correlated with environmental problem and food production, which has attracted extensive attention in the past several decades (Koops et al., 2006).

Nitrification is a vital microbial-mediated N cycling process in which ammonia (NH_3_) is converted to nitrite (NO_2_^–^) and nitrate (NO_3_^–^). Traditional wisdom holds that autotrophic nitrification is mainly carried out by two different groups of nitrifiers: ammonia-oxidizing bacteria (AOB) and archaea (AOA) (Prosser and Nicol, 2012), and nitrite-oxidizing bacteria (NOB), which consist of six genera, namely, *Nitrobacter*, *Nitrospira*, *Nitrococcus*, *Nitrotoga*, *Nitrolancetus*, and *Nitrospina* (Attard et al., 2010; Daims et al., 2011). In 2015, what is exciting is that researchers found that complete ammonia-oxidizing bacteria (Comammox) of the genus *Nitrospira* sublineage II (NOB) were capable of oxidizing NH_3_ to NO_3_^–^ in a single microorganism (Daims et al., 2015; van Kessel et al., 2015). A more recent study found that Comammox *Nitrospira* can be subdivided into two clades (A and B) based on the phylogeny of their ammonia monooxygenase (van Kessel et al., 2015). For decades, nitrification research has focused mainly on AOA and AOB, as they drive the first and often rate-limiting step of nitrification. In fact, nitrite oxidation can also become the limiting step for nitrification in disturbed soil ecosystems, and NOB has immense ecological significance as a principal source of NO_3_^–^ that supports primary biological production on Earth (Galloway et al., 2008).

The impacts of various fertilization measures on canonical ammonia oxidizers have been widely studied in terrestrial ecosystems (He et al., 2007; Di et al., 2009; Ouyang et al., 2018). A large number of studies have shown that AOB are more dominant in high NH_4_^+^-N environments, while AOA prefer low NH_4_^+^-N environments (Shen et al., 2008; Jia and Conrad, 2009; Di et al., 2010; Verhamme et al., 2011; Ouyang et al., 2017). In contrast, we have limited information about the response of NOB to chemical fertilization in the agricultural ecosystem. For Comammox *Nitrospira*, clades A and B present different responses to NH_4_^+^-N concentrations (Wang et al., 2019a). Comammox *Nitrospira* clade A was found to contribute to nitrification in the agricultural soils with high NH_4_^+^-N input (Li et al., 2019), while Comammox *Nitrospira* clade B as well as AOA played a pivotal role in soil nitrification in low NH_4_^+^-N or oligotrophic soil conditions (Wang et al., 2019b). The *Nitrobacter*- and *Nitrospira*-like NOB are believed to be the two key NOB groups in soils, and their responses to N fertilization are totally different. Previous studies found that *Nitrobacter*-like NOB was more responsive than *Nitrospira*-like NOB to high urea additions (Ke et al., 2013; Han et al., 2018). In addition, the rhizosphere and associated root exudates could be the hotspots of nutrient cycling in agricultural soil (Liu et al., 2018; Yu et al., 2019), but there is limited information available regarding the impacts of N fertilizer on nitrification and related nitrifiers in rhizosphere soil.

Biochar is commonly used as a soil amendment to improve soil fertility (Novak et al., 2009), increases crop yields (Lehmann, 2007), reduces greenhouse gas (Cayuela et al., 2014), and enhances soil carbon sequestration (Lehmann and Joseph, 2015). Biochar improves soil quality due to its effects on soil physicochemical properties, including enhancement of water- and nutrient-holding capacities, soil pH, organic C content, and cation exchange capacity (Pandit et al., 2018). Besides, previous studies have indicated that biochar addition has a significant impact on soil nitrification process and canonical ammonia oxidizers, although across different soils the effect can be stimulating (Teutscherova et al., 2017; He et al., 2018; Shi et al., 2019), inhibiting (Wang et al., 2015; Li et al., 2020), or have no difference (He et al., 2016). For example, an increase in soil pH due to biochar input into soils usually elevates AOB abundance and nitrification rate in an acidic soil (He et al., 2018). Another study found that coconut husk biochar amendment enhanced nutrient retention by reducing nitrifier abundance and nitrification activity (Plaimart et al., 2020). He et al. (2016) observed that the application of biochar had no effect on nitrification activity and AOA and AOB communities in Cambosols with high pH. However, the impacts of the biochar on community abundance and structure of NOB are not yet clear, and a recent study found that the inhibitory effect of biochar amendment was linked with the abundance of Comammox *Nitrospira* in a highly acidic soil (Li et al., 2020). In addition, previous studies just concentrated on assessing the impact of biochar addition on canonical ammonia oxidizers in bulk soils, but little research attention has been given to investigate the effects of biochar amendment on root-associated nitrifying communities.

In the present study, a field trial was conducted to study the influence of biochar and/or NPK additions on soil potential nitrification rate (PNR), community abundances, and structures of nitrifiers in rhizosphere soils of maize using a shaken-slurry procedure, real-time PCR, and terminal restriction fragment length polymorphism (T-RFLP). The objective of this research was to: (1) identify the responses of PNR and nitrifying community abundances and structures to biochar and/or NPK additions in rhizosphere soil, as well as their relationships with soil chemical properties; and (2) to evaluate the role of canonical ammonia oxidizers and nitrite oxidizers on PNR in rhizosphere soil. We hypothesized that: (1) combined application of biochar and/or chemical fertilizer would stimulate soil nitrification rate in the tested soil; and (2) soil properties including pH and nutrient contents in rhizosphere soil disturbed by biochar and/or chemical fertilizer addition would directly influence the community abundances and structures of nitrifiers.

## Materials and Methods

### Site Description and Experimental Design

The field site was located in Wengyuan County, Guangdong Province, China (24°35′N, 114°13′E), where the conventional farming system is maize (*Zea mays* L.) monoculture. This area has a subtropical monsoon climate, with an average annual temperature of 20.6°C and average annual precipitation of 1,778.8 mm. The soil is classified as Ferralic Cambisol, which is widespread in South China. A local high-yielding variety of maize (Yedan 13) was used in this experiment.

The field experiment was conducted on July 2017. Briefly, the experiment consisted of four treatments with three replicates. The four treatments in the plot were as follows: (1) without fertilizer (CK); (2) soil with biochar at 20 t ha^–1^ without fertilizer (B); (3) chemical nitrogen, phosphorus, and potassium fertilizers (NPK); and (4) biochar (20 t ha^–1^) plus NPK (B + NPK). Each field plot was 30 m^2^ (6 m × 5 m), and a 1-m buffer was set between adjacent plots. The biochar used in this study derived from sugarcane straw, which was pyrolyzed to biochar at 550°C. Its basic properties are according to Li et al. (2020). Chemical fertilizers were applied in the form of urea, a fusion of calcium/magnesium phosphate, and potassium chloride, respectively. The N fertilizer was applied at the rate of 250 kg ha^–1^ year^–1^ according to local conventional fertilization management. Approximately 40% of N was applied as basal fertilizer, and the remaining 60% of N was applied as topdressing fertilizer. All P and K fertilizers and biochar were applied as basal fertilizers at the rate of 90 kg ha^–1^ year^–1^, 90 kg ha^–1^ year^–1^, and 20 t ha^–1^, respectively. Biochar and inorganic fertilizers were uniformly applied to the topsoil and immediately plowed by tillage before sowing.

### Soil Sampling and Chemical Analysis

Soil samples were collected at the maize reproductive stage on October 2019, when the rhizosphere effects tend to be the most activated (Cheng et al., 2003). The rhizosphere sampled at the 0–20-cm depth from 10 maize plants were taken from each plot. The 10 samples from a given plot were then mixed to form a composite sample. Rhizosphere soil samples were taken by carefully picking up the maize root, gently shaking it, and then brushing the soil attached to the root into a collection bag (Wang et al., 2017). The samples were mixed thoroughly and sieved through a 2-mm sieve. One part of each soil sample was stored at −80°C for DNA extraction and molecular biology analyses, and the remaining sample was stored at 4°C for PNR and chemical property analyses.

All soil parameters were analyzed according to the methods of Lu (2000). Fresh soil samples were analyzed for soil nitrate-N (NO_3_^–^-N) and ammonium-N (NH_4_^+^-N). The fresh soils were air-dried for measuring soil pH, organic matter (OM), total nitrogen (TN), total phosphorus (TP), and available phosphorus (AP). Soil pH was measured with a water:soil ratio of 2.5 using a pH meter. OM was determined using the K_2_Cr_2_O_7_ oxidation method. TN was assessed using the Kjeldahl method. TP was digested by H_2_SO_4_/HClO_4_ and measured by the molybdenum blue method. AP in soil was extracted with ammonium fluoride-hydrochloric acid and assayed by molybdenum–antimony anti-colorimetric method. NH_4_^+^-N and NO_3_^–^-N were extracted with 2 M KCl solution and then determined on a San^++^ Continuous Flow Analyzer.

### Soil Potential Nitrification Rate Analysis

Soil PNR was measured using a shaken soil-slurry method with supplemental NH_4_^+^ (Kong et al., 2019). Briefly, 15 g of fresh soil was mixed with 100 ml buffer solution (1.0 mM NH_4_^+^, 1.0 mM PO_3_^–^, pH = 7.2) in a 250-ml bottle and then shaken at 150 rpm for 48 h at 25°C. Ten milliliters of suspension was taken at 0-, 24-, and 48-h intervals, respectively. The resulting supernatant was centrifuged at 10,000 rpm for 5 min, and the supernatant was analyzed for NO_3_^–^-N using the San^++^ Continuous Flow Analyzer.

### Soil DNA Extraction and Real-Time PCR

Soil DNA was extracted from 0.25 g fresh soil using MoBio PowerSoil DNA isolation Kit (MoBio Laboratory, Carlsbad, CA, United States). The quality and integrity of total DNA were assessed by 1% agarose gel electrophoresis. Quantification of functional marker genes (AOA, AOB, and Comammox *Nitrospira* clade A *amoA*, *Nitrobacter*-like *nxrA*, and *Nitrospira*-like *nxrB*) was carried out by quantitative PCR using a CFX96 Optical Real-Time Detection System (Bio-Rad, United States). The primer pairs and amplification procedures for quantification of functional genes are listed in Supplementary Table 1. The SYBR Premix Ex Taq^TM^ Kit (TaKaRa Biotechnology Co., Dalian, China) was used to conduct quantitative PCR reactions. The PCR reaction (25 μl total) contained 12.5 μl 2 × SYBR Premix Ex Taq^TM^ (Takara, Dalian), 0.5 μl of each primer, 2 μl DNA template (1–10 ng), and 9.5 μl dd H_2_O. Standard curves were obtained using plasmid DNA containing right inserts of the functional genes. Specific amplification was checked through a melting curve and a 1.5% agarose gel. However, we failed to amplify the band of Comammox *Nitrospira* clade B according to the gel electrophoresis detection system, and thus Comammox *Nitrospira* clade B was excluded from further analyses. The amplification efficiencies resulted in values of 88–95% for all functional genes, and the *r*^2^ values were > 0.99.

### Terminal Restriction Fragment Length Polymorphism Analysis of Nitrifying Community Structure

The AOA, AOB, Comammox *Nitrospira*, and *Nitrobacter*- and *Nitrospira*-like NOB community structures were analyzed by T-RFLP using the fluorescently labeled (6-FAM) forward primer (Ke et al., 2013; Hu et al., 2015). The PCR primer pairs and amplification conditions for these functional genes are shown in Supplementary Table 1. Each 50-μl reaction mixture consisted of 25 μl 2 × Premix (TaKaRa), 1, 10 μM forward and reverse primers, and 2 μl DNA template (10 ng). The labeled PCR products were separated by 1.5% agarose gel electrophoresis. After purification, the PCR products were then digested with restriction enzymes listed in Supplementary Table 2. The size of the terminal restriction fragments (T-RFs) and relative abundance were analyzed by an ABI 3730XL DNA analyzer (Applied Biosystems, Carlsbad, United States). GeneMarker 2.2 software was used to analyze T-RFLP profiles, and only peak heights > 100 fluorescence units were selected for the next step analysis. The relative abundance of each T-RF was calculated as the peak height percentage of a specific T-RF in the whole T-RFLP profile.

### Clone, Sequencing, and Phylogenetic Analysis

Clone libraries of AOA, AOB, Comammox *amoA* genes, and *Nitrobacter*-like *nxrA* and *Nitrospira*-like *nxrB* genes were constructed using the primers and reaction conditions in Supplementary Table 2. PCR products from each treatment were purified and ligated into the pMD19-T vector and transformed into competent *E. coli* top 10 cells. Positive clones for each functional gene were randomly selected for sequencing using an ABI 3730 sequencer (Applied Biosystems). Sequences in each clone library and the most similar sequences obtained from GenBank were used to construct the phylogenetic analysis. The phylogenetic analysis was conducted to construct a neighbor-joining tree using the Kimura two-parameter distance with 1,000 replicates to produce bootstrap values within MEGA 6.0. In order to identify the T-RFs of nitrifiers (AOA, AOB, Comammox, *Nitrobacter*, *Nitrospira*), clone libraries for each functional gene were constructed using the same primers as T-RFLP without the 6-FAM label. Virtual digests with restriction enzyme were carried out on the sequences retrieved from the clone libraries to allow the assignment of phylogenetic identify to individual T-RF. The obtained DNA sequences were deposited at the GenBank database under the following accession numbers: MZ614254–MZ614291 for AOA *amoA* gene, MZ614368–MZ614403 for AOB *amoA* gene, MZ614292–MZ614317 for Comammox *Nitrospira amoA* gene, MZ614318–MZ614339 for *Nitrobacter*-like *nxrA* gene, and MZ614340–MZ614367 for *Nitrospira*-like *nxrB* gene.

### Statistical Analyses

The effects of different treatments on soil chemical properties, PNR, the abundances of AOB, AOA, Comammox *Nitrospira* clade A, and *Nitrobacter*- and *Nitrospira*-like NOB were analyzed by one-way analysis of variance (ANOVA) based on the Duncan test with SPSS 20.0. Pearson correlation analysis was conducted to assess the relationships between soil chemical properties, PNR, and abundances of AOB, AOA, Comammox *Nitrospira* clade A, and *Nitrobacter*- and *Nitrospira*-like NOB using “corrplot” package in R software. Principal coordinate analysis (PCoA) was used to analyze the differences in nitrifying communities among the various treatments. Permutational multivariate analysis of variance (PERMANOVA) was used to test the differences in community structure among the different treatments. The detrended correspondence analysis (DCA) was performed by Canoco 5.0 to determine the overall functional changes of the nitrifying communities and found that the lengths of gradient for AOA, AOB, Comammox, *nxrA*, and *nxrB* were 0.3, 0.86, 0.28, 0.67, and 0.2, respectively. Therefore, redundancy analysis (RDA) was conducted to investigate the relationships between soil chemical properties and nitrifier community structure. Monte Carlo permutation test with 999 unrestricted permutations was used to determine the extent of the environmental parameter(s) that were able to explain the variation in the nitrifying communities. A random forest model was conducted using the “randomforest” and “A3” packages in R to select the main predictors of PNR. The mean square error (MSE) was calculated for each tree and averaged across 5,000 trees.

## Results

### Soil Chemical Properties and Potential Nitrification Rate

The effects of biochar and/or NPK addition on soil properties were analyzed (Table 1). Soil pH values ranged between 5.99 and 6.49 across the four different treatments. Compared with CK (pH = 5.99), biochar and/or NPK additions significantly increased soil pH (*p* < 0.05), with the highest value in the B treatment, which remained around 6.49 in rhizosphere soil. Similarly, compared with CK, biochar and/or NPK amendment obviously increased the contents of soil OM, AP, TP, TN, and NO_3_^–^ in rhizosphere soil, with the highest values in the B and B + NPK treatments. The highest concentration of NH_4_^+^ was found in the soil treated with NPK alone, followed by the B + NPK treatment, which was much higher than the CK treatment.

**TABLE 1:** Chemical properties of rhizosphere soil under different treatments.

Treatments	pH	OM (g kg^–1^)	AP (mg kg^–1^)	TP (g kg^–1^)	TN (g kg^–1^)	NO_3_^–^ (mg kg^–1^)	**NH_4_^+^ (mg kg^–1^)**
CK	5.99 ± 0.02c	15.1 ± 0.16d	181.17 ± 1.55c	0.45 ± 0.01c	0.64 ± 0.02c	3.03 ± 0.12c	4.37 ± 0.30c
B	6.46 ± 0.04a	24.21 ± 0.08b	244.55 ± 0.99a	0.72 ± 0.01a	1.32 ± 0.01a	7.91 ± 0.18b	4.06 ± 0.09c
NPK	6.12 ± 0.01b	19.29 ± 0.21c	237.8 ± 1.11b	0.58 ± 0.01b	0.87 ± 0.01b	7.35 ± 0.26b	125 ± 1.78a
B + NPK	6.49 ± 0.01a	28.87 ± 0.18a	242.24 ± 1.52a	0.69 ± 0.02a	1.31 ± 0.01a	15.5 ± 0.62a	79.83 ± 4.10b

*Means ± standard error, n = 3, different letters indicate significant differences among different treatments by one-way ANOVA (p < 0.05).*

*Treatments: CK, no fertilizer; B, biochar; NPK, nitrogen, phosphate, and potassium fertilizers; B + NPK, biochar + NPK.*

*OM, soil organic matter; AP, available P; TP, total P; TN, total N; NO_3_^–^, nitrate-N; NH_4_^+^, ammonium-N.*

**FIGURE 1:**
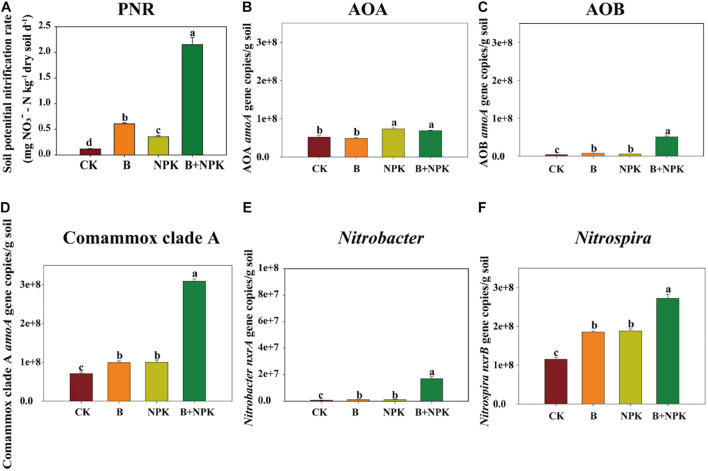
Potential nitrification activities (PNR) **(A)**; ammonia-oxidizing archaea (AOA) **(B)**, bacteria (AOB) **(C)**, and Comammox *Nitrospira* clade A **(D)** *amoA* gene abundances; *Nitrobacte*r-like *nxrA* **(E)** and *Nitrospira*-like *nxrB* **(F)** gene abundances in rhizosphere soil. Error bars represent standard errors (*n* = 3). Different letters indicate significant differences across different treatments (*p* < 0.05). Treatments: CK, no fertilizer; B, biochar; NPK, nitrogen, phosphate, and potassium fertilizers; B + NPK, biochar + NPK.

The soil PNR had a different response to biochar and/or NPK amendment (Figure 1A). The PNR was 0.13 mg NO_3_^–^-N kg^–1^ day^–1^ in the CK treatment, while B, NPK, and B + NPK treatments significantly increased PNR by 4.08, 2.00, and 16.92 times, respectively.

### Abundances of Nitrifying Communities

The abundances of AOA, AOB, Comammox *Nitrospira* clade A *amoA*, *Nitrobacter*-like NOB *nxrA*, and *Nitrospira*-like NOB *nxrB* genes were measured using real-time PCR assay. AOA abundance ranged from 4.84 × 10^7^ to 7.34 × 10^7^ copies g^–1^ soil in rhizosphere soil (Figure 1B). Compared to the CK treatment, NPK and B + NPK treatments showed significantly elevated AOA abundance (*p* < 0.05), while the B treatment alone had no effect on it (*p* > 0.05). The abundances of AOB ranged from 3.75 × 10^6^ to 5.10 × 10^7^ copies g^–1^ soil and the abundance of Comammox *Nitrospira* clade A ranged from 7.12 × 10^7^ to 3.09 × 10^8^ copies g^–1^ soil (Figures 1C,D). Biochar and/or NPK additions significantly increased the abundances of AOB and Comammox *Nitrospira* clade A (*p* < 0.05), with the highest values (5.10 × 10^7^ and 3.09 × 10^8^ copies g^–1^ soil, respectively) in the B + NPK treatment (*p* < 0.01).

*Nitrobacter*- and *Nitrospira*-like NOB abundances ranged from 7.17 × 10^5^ to 2.32 × 10^7^ copies g^–1^ soil and 1.15 × 10^8^ to 2.72 × 10^8^ copies g^–1^ soil, respectively (Figures 1E,F). The highest *Nitrobacter*- and *Nitrospira*-like NOB abundances were detected in the B + NPK treatment, while the lowest values were observed in the CK treatment. The abundances of *Nitrobacter*- and *Nitrospira*-like NOB in the NPK and B treatments were higher than those in the CK treatment.

### Community Structure of Canonical Ammonia Oxidizers and Nitrite Oxidizers Based on Terminal Restriction Fragment Length Polymorphism

**FIGURE 2:**
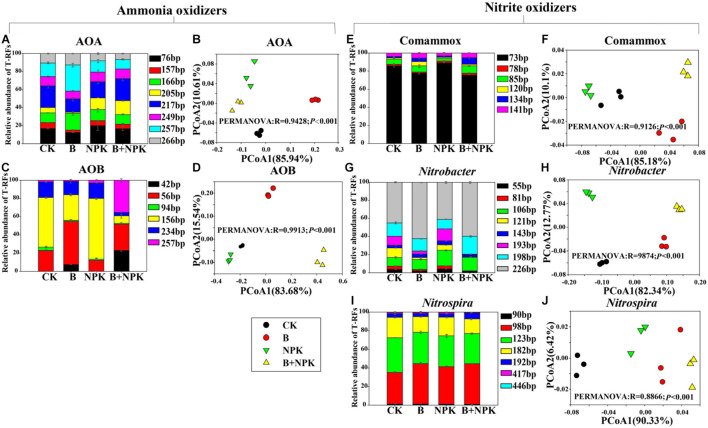
Treatment effects on nitrifying communities in rhizosphere soil characterized by terminal restriction fragment length polymorphism (T-RFLP). The T-RFLP profiles and principal coordinate analysis (PCoA) graphs of ammonia-oxidizing archaea (AOA) *amoA* **(A,B)**, ammonia-oxidizing bacteria (AOB) *amoA* **(C,D)**, Comammox *Nitrospira amoA* **(E,F)**, *Nitrobacter*-like *nxrA* **(G,H)**, and *Nitrospira*-like *nxrB* **(I,J)** genes across treatments. Permutational multivariate analysis of variance (PERMANOVA) was used to test the differences in community structure among the different treatments. Treatments: CK, no fertilizer; B, biochar; NPK, nitrogen, phosphate, and potassium fertilizers; B + NPK, biochar + NPK. OM, soil organic matter; AP, available P; TP, total P; TN, total N; NO_3_^–^, nitrate-N; NH_4_^+^, ammonium-N.

The PCoA of AOA, AOB, Comammox, *Nitrobacter*-like NOB, and *Nitrospira*-like NOB community structure revealed that the first two axes explained 96.55, 99.22, 95.28, 95.11, and 96.75% of the total variance, respectively (Figures 2B,D,F,H,J). In addition, the community structure of these nitrifiers under CK treatment was significantly separated from those in the other fertilization treatments (B, NPK, B + NPK), which was further supported by PERMANOVA analysis (Figures 2B,D,F,H,J). These results indicated that biochar and/or chemical fertilization changed the nitrifier community structure.

Biochar and/or NPK addition significantly affected the relative abundances of the major canonical ammonia oxidizers and nitrite oxidizers as indicated by T-RFLP analysis (Figure 2). Eight major T-RFs were obtained from the T-RFLP profile of the AOA *amoA* gene using the enzyme *Hpy*CH4V (Figure 2A). The relative abundance of T-RF 76 bp was decreased by single biochar addition, while it was increased by single NPK amendment. However, the application of combined biochar with NPK (B + NPK) had little impact on its relative abundance compared with the CK treatment in rhizosphere soil. Biochar and/or NPK addition significantly reduced the relative abundances of T-RFs by 157, 205, and 249 bp. The relative abundance of T-RF 166 bp was enhanced in the B treatment but decreased in the NPK and B + NPK treatments. The relative abundance of 217 bp was decreased in the B treatment compared with CK treatment, while NPK and B + NPK treatments did not influence it. The relative abundance of T-RF 257 bp was increased in the B and B + NPK treatments, while NPK treatment showed no effect on it, compared with CK treatment. AOA *amoA* sequences obtained from the clone library were assigned to *Nitrosotalea* and *Nitrososphaera* clusters (Supplementary Figure 1). The T-RF 76 bp was affiliated with both *Nitrosotalea* and *Nitrososphaera* clusters. The T-RFs of 166, 205, and 249 bp were assigned to *Nitrosotalea*, while the T-RFs of 217 and 257 bp belonged to *Nitrososphaera*.

For AOB, there were also six main T-RFs reflecting the relative abundance using the enzyme *Msp*I (Figure 2C). The relative abundance of T-RF 42 bp was not detected in the CK and NPK treatments, but it was significantly increased in the B and B + NPK treatments. The B and B + NPK treatments significantly elevated the relative abundance of T-RF 56 bp, whereas this T-RF decreased in the NPK treatment. The relative abundance of T-RFs 94 bp was significantly decreased in the B, NPK, and B + NPK treatments. The relative abundance of T-RF 156 bp was significantly decreased in the B and B + NPK treatments but significantly increased in the NPK treatment. The relative abundance of T-RF 234 bp was significantly decreased in the B and B + NPK treatments. The relative abundance of T-RF 257 bp was significantly increased in the NPK and B + NPK treatments. AOB *amoA* sequences obtained from the clone library were assigned to *Nitrosospira* and *Nitrosomonas* (Supplementary Figure 2). The T-RF 56 bp was affiliated with both *Nitrosospira* and *Nitrosomonas*. The T-RFs of 156, 234, and 257 bp were assigned to *Nitrosospira*, while the T-RFs of 217 and 257 bp belonged to *Nitrososphaera*. Six main T-RFs were obtained from the T-RFLP profile of Comammox *Nitrospira amoA* across all the soil samples (Figure 2E). The relative abundance of T-RF 73 bp was significantly decreased in the B and B + NPK treatments compared with the CK treatment, while the relative abundances of T-RFs 85, 134, and 141 bp were significantly increased. The relative abundance of T-RF 85 bp was significantly decreased in the NPK treatment while the relative abundance of T-RF 121 bp significantly increased in the B treatment, compared with the CK treatment. However, biochar and/or NPK addition did not affect the relative abundance of T-RF 78 bp. The phylogenetic analysis showed that all Comammox *amoA* sequences were assigned to Comammox clade A (Supplementary Figure 3). Eight and six major T-RFs were obtained from the T-RFLP profile of *Nitrobacter*-like *nxrA* and *Nitrospira*-like *nxrB* genes across soil samples, respectively (Figures 2G,I). For *Nitrobacter*-like NOB, the relative abundance of T-RF 266 bp was the dominant *Nitrobacter*-like *nxrA* genotype and significantly increased in the B and B + NPK treatments (Figure 2G). Besides, the relative abundances of T-RFs 55, 81, 121, and 193 bp were significantly decreased in the B and B + NPK treatments, while NPK treatment had no impact on them. The relative abundance of T-RF 106 bp was significantly increased in the NPK and B + NPK treatments, compared with the CK treatment. The relative abundance of T-RF 198 bp was only significantly increased in the B + NPK treatment. The phylogenetic analysis showed that the T-RFs of 55, 106, and 198 bp were only affiliated with *Nitrobacter alkalicus*, while T-RF 226 bp was assigned to both *Nitrobacter alkalicus* and *Nitrobacter winogradskyi* (Supplementary Figure 4). For *Nitrospira*-like NOB, the relative abundances of T-RF 98, 123, 182, and 192 bp were major fragments of the *Nitrospira*-like NOB *nxrB* gene (Figure 2I). The relative abundance of T-RF 98 bp in rhizosphere soil was significantly increased in B, NPK, and B + NPK treatments, while the relative abundances of T-RFs 123 and 182 bp were significantly decreased. The relative abundance of T-RF 192 bp was significantly increased in the B + NPK treatment, while single biochar or NPK addition had little impact on it. The phylogenetic analysis showed that T-RF 98 bp was assigned to *Nitrospira* lineage I and II. T-RF 123 bp was affiliated with Namibia soil cluster, *Nitrospira* lineage V and VI, while T-RF 182 bp belonged to *Nitrospira* lineage II and V.

### Relationships Between Nitrifiers and Soil Chemical Properties and Predictive Importance of Nitrifiers to Nitrification

**FIGURE 3:**
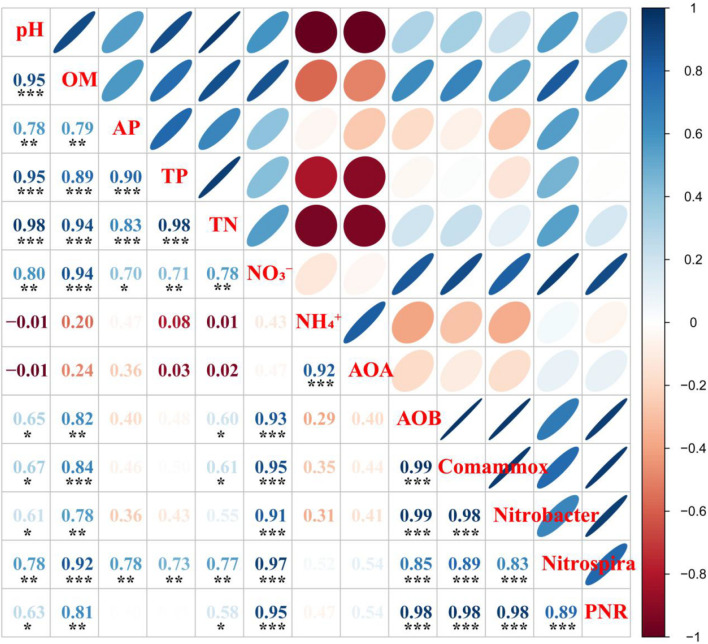
Pearson correlation analyses of soil chemical properties and potential nitrification rate (PNR), and nitrifying community abundances in rhizosphere soil. Blue denotes the significance level *p* < 0.05, the smaller shapes and darker color denote the larger correlation coefficients; red denotes the significance level *p* > 0.05, the larger shapes and darker color denote the smaller correlation coefficients. The correlation coefficients are shown in the lower left panel, * indicates *p* < 0.05, ** indicates *p* < 0.01, and *** indicates *p* < 0.001. OM, soil organic matter; AP, available P; TP, total P; TN, total N; NO_3_^–^, nitrate-N; NH_4_^+^, ammonium-N; AOA, ammonia-oxidizing archaea; AOB, ammonia-oxidizing bacteria; Comammox *Nitrospira*, complete ammonia oxidizers; *Nitrobacter*, *Nitrobacter*-like NOB; *Nitrospira*, *Nitrospira*-like NOB.

As shown in Figure 3, Pearson correlation analyses indicated that AOA abundance was positively correlated with NH_4_^+^ (*p* < 0.001), while AOB and Comammox *Nitrospira* clade A abundances were positively correlated with pH, OM, TN, and NO_3_^–^ (*p* < 0.05 or *p* < 0.001). The abundance of *Nitrobacter*-like NOB was positively correlated with pH, OM, and NO_3_^–^ (*p* < 0.05, *p* < 0.01, or *p* < 0.001) while the abundance of *Nitrospira*-like NOB was positively correlated with pH, OM, AP, TP, TN, and NO_3_^–^ (*p* < 0.01 or *p* < 0.001).

**FIGURE 4:**
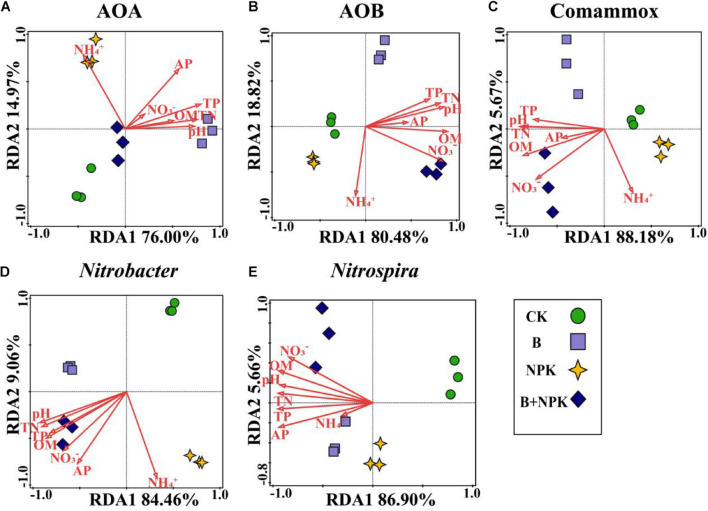
Redundancy analysis (RDA) evaluates the relationships between soil chemical properties and nitrifying communities in rhizosphere soil. **(A)** Ammonia-oxidizing archaea (AOA), **(B)** ammonia-oxidizing bacteria (AOB), **(C)** Comammox *Nitrospira*, **(D)** *Nitrobacter*-like NOB, and **(E)** *Nitrospira*-like NOB. Treatments: CK, no fertilizer; B, biochar; NPK, nitrogen, phosphate, and potassium fertilizers; B + NPK, biochar + NPK. OM, soil organic matter; AP, available P; TP, total P, TN: total N; NO_3_^–^, nitrate-N; NH_4_^+^, ammonium-N.

The correlations between soil properties and nitrifiers community structure were assessed by RDA (Figure 4). Overall, the first two axes explained 92.47, 98.63, 94.10, 93.54, and 93.60% of the total variability in the community structures of AOA, AOB, Comammox *Nitrospira*, *Nitrobacter*-, and *Nitrospira*-like NOB, respectively. Among the multiple soil variables, pH and most nutrient parameters were the primary factors influencing nitrifier community structure (Figures 4A–D).

**FIGURE 5:**
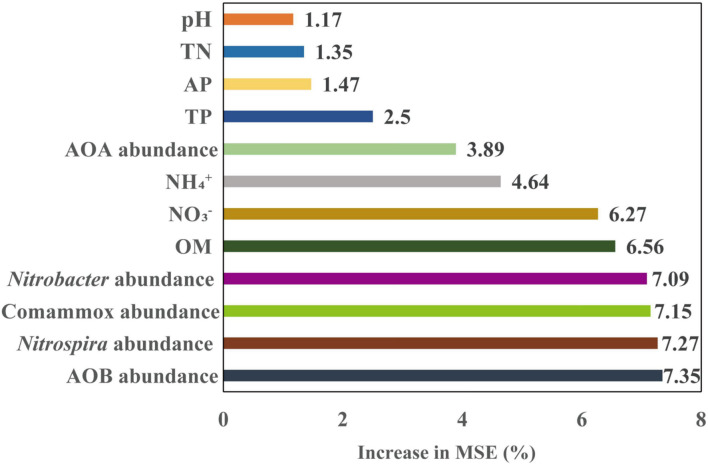
Random forest model predicts the relative importance of soil chemical properties and abundances of nitrifying communities, as measured by percentage of increase in mean square error (MSE) of the potential nitrification rate (PNR). OM, soil organic matter; AP, available P; TP, total P; TN, total N; NO_3_^–^, nitrate-N; NH_4_^+^, ammonium-N; AOA, ammonia-oxidizing archaea; AOB, ammonia-oxidizing bacteria; Comammox *Nitrospira*, complete ammonia oxidizers; *Nitrobacter*, *Nitrobacter*-like NOB; *Nitrospira*, *Nitrospira*-like NOB.

Soil PNR was significantly correlated with soil pH, OM, TN, and NO_3_^–^ (*p* < 0.05, *p* < 0.01, or *p* < 0.001) (Figure 3). In addition, soil PNR was significantly and positively correlated with the abundances of AOB, Comammox *Nitrospira* clade A, and *Nitrobacter*-like, and *Nitrospira*-like NOB (Figure 3). Random forest analysis was conducted to reveal the main factors in predicting the PNR in the study. *Nitrospira*-like NOB, AOB, Comammox *Nitrospira* clade A, and *Nitrobacter*-like NOB abundances were the major predictors for PNR (Figure 5). In addition, PNR was also predicted by OM and NO_3_^–^, which was consistent with Pearson’s correlation analysis (Figures 3, 5).

## Discussion

### Effects of Biochar and/or NPK Amendment on Potential Nitrification Rate in Rhizosphere Soil

In this study, we characterized the effect of biochar and/or NPK fertilizer additions on PNR to better understand how these measures influenced the soil nitrification process. PNR was very weak in the CK treatment, while NPK addition alone significantly increased it, probably due to the absence of substrate NH_4_^+^ (He et al., 2016). Addition of biochar alone also significantly stimulated soil PNR compared to the CK treatment, indicating that biochar addition had a positive effect on PNR, which was in line with a recent study that manure-based biochar increased the gross nitrification rate in rhizosphere soil using the ^15^N isotope labeling technique (Pokharel et al., 2021). Moreover, PNR in the B + NPK treatment was observed higher than in the NPK treatment, demonstrating that application of combined biochar with NPK had a synergistic effect on soil nitrification. Biochar may significantly improve soil structure and aeration due to its high porosity and low bulk density (Joseph et al., 2010), thereby providing a suitable environment for a high nitrification rate. In addition, biochar elevates soil organic matter, leading to a high C/N ratio that could stimulate the soil nitrification rate (Bi et al., 2017). Furthermore, biochar is usually alkaline and its application could increase soil pH, another factor that could promote soil nitrification (Cayuela et al., 2014). In our study, Pearson correlation analysis showed a positive relationship between PNR and pH and nutrient contents including OM and TN. Overall, these results supported our hypothesis that biochar and/or NPK addition had positive effects on PNR as they elevate soil pH and nutrient availability.

### Effects of Biochar and/or NPK Amendment on Abundances and Community Structures of Canonical Ammonia Oxidizers in Rhizosphere Soil

Canonical ammonia oxidizers (AOA and AOB) carry out the first step of nitrification, in which ammonia is converted to nitrite by encoding the subunit of ammonia monooxygenase, the limiting factor in soil nitrification (Shi et al., 2019). In agreement with previous studies (Leininger et al., 2006; He et al., 2018; Yu et al., 2019), AOA dominated in abundance compared to AOB in the tested soil. Single biochar amendment had little impact on AOA abundance, which was contrary to a previous study (Xiao et al., 2019). The higher AOA abundance in NPK and B + NPK treatments compares with CK, indicating that substrate NH_4_^+^ concentration may be the limiting factor for AOA growth in the selected soil. The positive correlation between AOA and NH_4_^+^ concentration supported our argument. Additionally, the abundance of AOB significantly enhanced in the biochar and/or NPK treatments, accompanied by higher nitrification rates in these soils. Meanwhile, a strong positive relationship was found between AOB abundance and soil PNR (*r* = 0.98, *p* < 0.001), which indicated that AOB might play a more important role than AOA in soil nitrification activity in biochar and/or NPK treatments. Particularly, the highest abundance of AOB and PNR was observed in biochar + NPK treatment, suggesting that the application of combined biochar and NPK could increase nitrification rates by increasing AOB abundance in rhizosphere soil. Consistent with our findings, He et al. (2018) showed that the application of biochar increased AOB abundance and PNR in oxisols with low pH compared with CK. Another study also reported that AOB abundance was strongly correlated with net nitrification rate and that AOB abundance followed a similar trend as NO_3_^–^ in biochar amendment treatments under a microcosm experiment, implying that AOB could contribute more to nitrification in degraded acid soil when biochar and N fertilizer is added together (Teutscherova et al., 2017). A large number of studies have indicated that the application of biochar would provide a better living environment by increasing soil pH (liming effect) (Dai et al., 2016), heighten nutrient retention and soil fertility (Hagemann et al., 2017), and improve soil aeration (Cayuela et al., 2014), and these factors are often linked with the growth of AOA and AOB (Xiao et al., 2019).

The T-RFLP analysis showed that single biochar amendment significantly altered the community structures of canonical ammonia oxidizers in the rhizosphere soil. This result differs from a previous study that showed single biochar addition did not significantly change AOA and AOB community structures in an intensive vegetable soil (Bi et al., 2017). In this case, we suspect that the effect of biochar addition on community structures of canonical ammonia oxidizers strongly depends on soil type, with fundamental soil properties affecting the effect of biochar. In addition, this study also found that the community structures of AOA and AOB were significantly altered by NPK amendment, which was in line with a previous study that the application of inorganic fertilizer containing N showed significant impact on AOA and AOB community structures in acidic red soil (He et al., 2007). Furthermore, the community structures of AOA and AOB were significantly shifted by the combined application of biochar and NPK, which partly aligns with previous studies showing that combined application of biochar with N fertilizer influenced AOB but not AOA community structure (Bi et al., 2017; Lin et al., 2017). Based on the RDA analysis, the reason that significant changes in community structures of canonical ammonia oxidizers were observed in our study could be elevated pH and nutrient contents after biochar and/or NPK amendment.

### Effects of Biochar and/or NPK Amendment on Abundances and Community Structures of Nitrite Oxidizers in Rhizosphere Soil

In our study, we found that Comammox *Nitrospira* clade A outnumbered canonical ammonia oxidizers (AOA and AOB). Similar results were also reported in a subtropical paddy soil (Liu et al., 2019), pasture soil (Li et al., 2019), and forest soil (Hu and He, 2017; Osburn and Barrett, 2020). A higher abundance of Comammox *Nitrospira* has been observed in environments with low NH_3_ concentration (Hu and He, 2017), and *Nitrospira inopinata*, the only pure strain of Comammox bacteria, is assumed to have an oligotrophic lifestyle in a previous study (Kits et al., 2017). However, in contrast to traditional understanding, we found that the application of NPK or combined biochar and NPK (i.e., eutrophic conditions) significantly elevated Comammox *Nitrospira* clade A abundance, which was significantly and positively correlated with soil nitrification rate. These results provide new evidence that Comammox *Nitrospira* clade A may play an active role in nitrification of tested rhizosphere soil-amended biochar and/or NPK fertilizer. Single biochar addition significantly increased *Nitrobacter*-like NOB rather than *Nitrospira*-like NOB abundance, indicating that biochar showed a positive effect on it. Previous studies reported that *Nitrobacter*-like NOB had a lower affinity than *Nitrospira*-like NOB for N substrate and could be stimulated by high N levels (Wagner et al., 2002; Attard et al., 2010; Ke et al., 2013; Han et al., 2018). For example, Ma et al. (2016) found that *Nitrobacter*-like NOB abundance in a pasture soil elevated linearly with increasing N fertilizer amendment gradient while *Nitrospira*-like NOB was not obviously affected. Wertz et al. (2011) also observed that N fertilization enhanced *Nitrobacter*-like NOB abundance but not *Nitrospira*-like NOB in forest soils. However, in our study, the abundances of *Nitrobacter*- and *Nitrospira*-like NOB were both significantly increased in NPK treatment, which was also consistent with a recent study (Kong et al., 2019). Thus, the response of *Nitrospira*-like NOB to N fertilization is likely dependent on soil type or other complex ecosystem features and needs to be more thoroughly investigated in future. Furthermore, the higher abundances of *Nitrobacter*- and *Nitrospira*-like NOB were observed in B + NPK treatment compared with NPK alone, suggesting that biochar and NPK interact to exert a greater effect on NOB abundance. In our study, Pearson correlation analysis showed that changes in the abundances of Comammox *Nitrospira* clade A, *Nitrobacter*- and *Nitrospira*-like NOB were significantly and positively correlated with soil pH and multiple nutrient contents, suggesting that soil pH and nutrient availability may explain in part their promoting effect on the growth of nitrite oxidizers.

Our study showed that biochar and/or NPK amendment significantly changed the community structures of nitrite oxidizers (Comammox *Nitrospira*, and *Nitrobacter*- and *Nitrospira*-like NOB) in rhizosphere soil. To better understand the complex interaction of factors, it is necessary to explore how soil chemical properties influenced nitrite oxidizers community structures under biochar and/or NPK addition. The RDA analysis suggested that the variability of nitrite oxidizers was remarkably affected by soil pH and multiple nutrients (Figure 4), in agreement with recent studies (Han et al., 2018; Wang et al., 2019a; Yu et al., 2019). Wang et al. (2019a) found that pH, OM, and available N are key factors affecting Comammox community structure. Han et al. (2018) reported that *Nitrobacter*-like community structure was strongly influenced by pH and soil organic carbon content and the *Nitrospira*-like NOB community structure was significantly explained by pH and TN. Therefore, biochar and/or NPK amendment resulted in a shift of soil pH and nutrient availability, which are the key factors affecting community structures of nitrite oxidizers in the rhizosphere soil.

## Conclusion

Our present study revealed that biochar and/or NPK addition significantly increased the soil nitrification rate in rhizosphere soil of maize. Single biochar addition significantly increased AOB, Comammox *Nitrospira* clade A, *Nitrobacter*-like NOB and *Nitrospira*-like NOB abundances, while it showed no impact on AOA abundance. Biochar and/or NPK addition also changed nitrifying community structures, and pH and nutrient availability were key factors influencing them. We found positive correlations between AOB, Comammox *Nitrospira* clade A, and *Nitrobacter*- and *Nitrospira*-like NOB abundances and potential nitrification rates, indicating that these nitrifiers may play important roles in soil nitrification.

## Data Availability Statement

The original contributions presented in the study are included in the article/Supplementary Material, further inquiries can be directed to the corresponding author/s.

## Author Contributions

QW contributed to the design of the research and revised the manuscript. PS and ZZ conducted the experiment and participated in drafting the manuscript. PF, WC, and YR contributed to analyze the results. All authors have read and approved the final manuscript.

## Conflict of Interest

The authors declare that the research was conducted in the absence of any commercial or financial relationships that could be construed as a potential conflict of interest.

## Publisher’s Note

All claims expressed in this article are solely those of the authors and do not necessarily represent those of their affiliated organizations, or those of the publisher, the editors and the reviewers. Any product that may be evaluated in this article, or claim that may be made by its manufacturer, is not guaranteed or endorsed by the publisher.

## References

[B1] Attard E. Poly F. Commeaux C. Laurent F. Terada A. Smets B. F. (2010). Shifts between Nitrospira- and Nitrobacter-like nitrite oxidizers underlie the response of soil potential nitrite oxidation to changes in tillage practices. *Environ. Microbiol.* 12 315–326. 10.1111/j.1462-2920.2009.02070.x 19807778

[B2] Bi Q. F. Chen Q. H. Yang X. R. Li H. Zheng B. X. Zhou W. W. (2017). Effects of combined application of nitrogen fertilizer and biochar on the nitrification and ammonia oxidizers in an intensive vegetable soil. *Amb. Express* 7:198. 10.1186/s13568-017-0498-7 29116481 PMC5676586

[B3] Cayuela M. L. van Zwieten L. Singh B. P. Jeffery S. Roig A. Sánchez-Monedero M. A. (2014). Biochar’s role in mitigating soil nitrous oxide emissions: a review and meta-analysis. *Agri. Ecosyst. Environ.* 191 5–16. 10.1016/j.agee.2013.10.009

[B4] Cheng W. Johnson D. Fu S. (2003). Rhizosphere effects on decomposition: controls of plant species, phenology, and fertilization. *Soil Sci. Soc. America J.* 67 1418–1427. 10.2136/sssaj2003.1418

[B5] Dai Z. M. Zhang X. J. Tang C. Muhammad N. Wu J. J. Brookes P. C. (2016). Potential role of biochars in decreasing soil acidification-a critical review. *Sci. Total Environ.* 78 1601–1611. 10.1016/j.scitotenv.2016.12.169 28063658

[B6] Daims H. Lebedeva E. V. Pjevac P. Han P. Herbold C. Albertsen M. (2015). Complete nitrifification by Nitrospira bacteria. *Nature* 528 504–509. 10.1038/nature16461 26610024 PMC5152751

[B7] Daims H. Lücker S. Paslier D. L. Wagner M. (2011). “Diversity, environmental genomics, and ecophysiology of nitrite-oxidizing bacteria,” in *Nitrification*, eds Ward B. B. Arp D. J. Klotz M. G. (Washington, DC: ASM Press), 295–322.

[B8] Di H. J. Cameron K. C. He J. Z. Shen J. P. Winefield C. S. O’Callaghan M. (2010). Ammonia-oxidizing bacteria and archaea grow under contrasting soil nitrogen conditions. *FEMS Microbiol. Ecol.* 72 386–394. 10.1111/j.1574-6941.2010.00861.x 20370827

[B9] Di H. J. Cameron K. C. Shen J. P. Winefield C. S. O’Callaghan M. Bowatte S. (2009). Nitrification driven by bacteria and not archaea in nitrogen-rich grassland soils. *Nat. Geosci.* 2 621–624. 10.1038/ngeo613

[B10] Galloway J. N. Townsend A. R. Erisman J. W. Bekunda M. Cai Z. C. Freney J. R. (2008). Transformation of the nitrogen cycle: recent trends, questions, and potential solutions. *Science* 320 889–892. 10.1126/science.1136674 18487183

[B11] Guo J. H. Liu X. J. Zhang Y. Shen J. L. Han W. X. Zhang W. F. (2010). Significant acidification in major Chinese croplands. *Science* 327 1008–1010. 10.1126/science.1182570 20150447

[B12] Hagemann N. Joseph S. Schmidt H. P. Kammann C. I. Harter J. Borch T. (2017). Organic coating on biochar explains its nutrient retention and stimulation of soil fertility. *Nat. Commun.* 8:1089. 10.1038/s41467-017-01123-0 29057875 PMC5715018

[B13] Han S. Zeng L. Y. Luo X. S. Xiong X. Wen S. L. Wang B. R. (2018). Shifts in Nitrobacter- and Nitrospira-like nitrite-oxidizing bacterial communities under long-term fertilization practices. *Soil Biol. Biochem.* 124 118–125. 10.1016/j.soilbio.2018.05.033

[B14] He J. Z. Shen J. P. Zhang L. M. Zhu Y. G. Zheng Y. M. Xu M. G. (2007). Quantitative analyses of the abundance and composition of ammonia-oxidizing bacteria and ammonia-oxidizing archaea of a Chinese upland red soil under long-term fertilization practices. *Environ. Microbiol.* 9 2364–2374. 10.1111/j.1462-2920.2007.01481.x 17686032

[B15] He L. L. Bi Y. C. Zhao J. Pittelkow C. M. Zhao X. Wang S. Q. (2018). Population and community structure shifts of ammonia oxidizers after four-year successive biochar application to agricultural acidic and alkaline soils. *Sci. Total Environ.* 620 1105–1115. 10.1016/j.scitotenv.2017.11.029 29734589

[B16] He L. L. Liu Y. Zhao J. Bi Y. C. Zhao X. Wang S. Q. (2016). Comparison of straw-biochar-mediated changes in nitrification and ammonia oxidizers in agricultural oxisols and cambosols. *Biol. Fertil. Soils* 52 137–149. 10.1007/s00374-015-1059-3

[B17] Hu H. W. Chen D. L. He J. Z. (2015). Microbial regulation of terrestrial nitrous oxide formation: understanding the biological pathways for prediction of emission rates. *FEMS Microbiol. Rev.* 39 729–749. 10.1093/femsre/fuv021 25934121

[B18] Hu H. W. He J. Z. (2017). Comammox—a newly discovered nitrification process in the terrestrial nitrogen cycle. *J. Soils Sediments* 17 2709–2717. 10.1007/s11368-017-1851-9

[B19] Jia Z. J. Conrad R. (2009). Bacteria rather than archaea dominate microbial ammonia oxidation in an agricultural soil. *Environ. Microbiol.* 11 1658–1671. 10.1111/j.1462-2920.2009.01891.x 19236445

[B20] Joseph S. D. Camps-Arbestain M. Lin Y. Munroe P. Chia C. H. Hook J. (2010). An investigation into the reactions of biochar in soil. *Soil Res.* 487 501–515. 10.1071/SR10009

[B21] Ke X. Angel R. Lu Y. Conrad R. (2013). Niche differentiation of ammonia oxidizers and nitrite oxidizers in rice paddy soil. *Environ. Microbiol.* 15 2275–2292. 10.1111/1462-2920.12098 23437806

[B22] Kits K. D. Sedlacek C. J. Lebedeva E. V. Han P. Bulaev A. Pjevac P. (2017). Kinetic analysis of a complete nitrifier reveals an oligotrophic lifestyle. *Nature* 54 269–272. 10.1038/nature23679 28847001 PMC5600814

[B23] Kong Y. L. Ling N. Xue C. Chen H. Ruan Y. Guo J. J. (2019). Long-term fertilization regimes change soil nitrification potential by impacting active autotrophic ammonia oxidizers and nitrite oxidizers as assessed by DNA stable isotope probing. *Environ. Microbiol.* 21 1224–1240. 10.1038/nature23679 30724443

[B24] Koops H. P. Purkhold U. Pommerening-Röser A. Timmermann G. Wagner M. (2006). “The lithoautotrophic ammonia-oxidizing bacteria,” in *The Prokaryotes: Proteobacteria: Alpha and Beta Subclasses*, eds Dworkin M. Falkow S. Rosenberg E. Schleifer K.-H. Stackebrandt E. (New York, NY: Springer New York), 778–811.

[B25] Lehmann J. (2007). A handful of carbon. *Nature* 447 143–144. 10.1038/447143a 17495905

[B26] Lehmann J. Joseph S. (2015). *Biochar for Environmental Management: Science, Technology and Implementation*, 2nd Edn. London: Earthscan.

[B27] Leininger S. Urich T. Schloter M. Schwark L. Qi J. Nicol G. (2006). Archaea predominate among ammonia-oxidizing prokaryotes in soils. *Nature* 442 806–809. 10.1038/nature04983 16915287

[B28] Li C. Y. Hu H. W. Chen Q. L. Chen D. He J. Z. (2019). Comammox Nitrospira play an active role in nitrification of agricultural soils amended with nitrogen fertilizers. *Soil Biol. Biochem.* 138:107609. 10.1016/j.soilbio.2019.107609

[B29] Li S. Chen D. W. Wang C. Chen D. Wang Q. (2020). Reduced nitrification by biochar and/or nitrification inhibitor is closely linked with the abundance of Comammox Nitrospira in a highly acidic sugarcane soil. *Biol. Fertil. Soils* 56 1219–1228. 10.1007/s00374-020-01499-0

[B30] Lin Y. X. Ding W. X. Liu D. Y. He T. H. Yoo G. Y. Yuan J. J. (2017). Wheat straw-derived biochar amendment stimulated N2O emissions from rice paddy soils by regulating the amoA genes of ammonia-oxidizing bacteria. *Soil Biol. Biochem.* 113 89–98. 10.1016/j.soilbio.2017.06.001

[B31] Liu T. Wang Z. Wang S. Zhao Y. Wright A. L. Jiang X. (2019). Responses of ammonia-oxidizers and comammox to different long-term fertilization regimes in a subtropical paddy soil. *Europ. Soil Biol.* 93:103087. 10.1016/j.ejsobi.2019.103087

[B32] Liu X. R. Li J. Yu L. Pan H. Liu H. Y. Liu Y. M. (2018). Simultaneous measurement of bacterial abundance and composition in response to biochar in soybean field soil using 16S rRNA gene sequencing. *Land Degrad. Dev.* 29 2172–2182. 10.1002/ldr.2838

[B33] Liu Y. B. Pan X. B. Li J. S. (2015). A 1961-2010 record of fertilizer use, pesticide application and cereal yields: a review. *Agro. Sustainable Dev.* 35 83–93. 10.1007/s13593-014-0259-9

[B34] Lu R. K. (2000). *Soil Agro-Chemical Analyses.* Beijing: Agricultural Technical Press of China.

[B35] Ma W. B. Jiang S. J. Assemien F. E. L. Qin M. S. Ma B. B. Xie Z. (2016). Response of microbial functional groups involved in soil N cycle to N, P and NP fertilization in Tibetan alpine meadows. *Soil Biol. Biochem.* 101 195–206. 10.1016/j.soilbio.2016.07.023

[B36] Novak J. M. Bussche R. W. J. Laird D. L. Ahmedna M. Watts D. W. Niandou M. A. S. (2009). Impact of biochar amendment on fertility of a southeastern coastal plain soil. *Soil Sci.* 174 105–112. 10.1097/SS.0b013e3181981d9a

[B37] Osburn E. D. Barrett J. E. (2020). Abundance and functional importance of complete ammonia-oxidizing bacteria (comammox) versus canonical nitrifiers in temperate forest soils. *Soil Biol. Biochem.* 145:107801. 10.1016/j.soilbio.2020.107801

[B38] Ouyang Y. Evans S. E. Friesen M. L. Tiemann L. K. (2018). Effect of nitrogen fertilization on the abundance of nitrogen cycling genes in agricultural soils: a meta-analysis of field studies. *Soil Biol. Biochem.* 127 71–78. 10.1016/j.soilbio.2018.08.024

[B39] Ouyang Y. Norton J. M. Stark J. M. (2017). Ammonium availability and temperature control contributions of ammonia oxidizing bacteria and archaea to nitrification in an agricultural soil. *Soil Biol. Biochem.* 113 161–172. 10.1016/j.soilbio.2017.06.010

[B40] Pandit N. R. Mulder J. Hale S. E. Zimmerman A. R. Pandit B. H. Cornelissen G. (2018). Multi-year double cropping biochar field trials in Nepal: finding the optimal biochar dose through agronomic trials and cost-benefit analysis. *Sci. Total Environ.* 63 1333–1341. 10.1016/j.scitotenv.2018.05.107 29801225

[B41] Plaimart J. Acharya K. Mrozik W. Davenport R. J. Vinitnantharat S. Werner D. (2020). Coconut husk biochar amendment enhances nutrient retention by suppressing nitrification in agricultural soil following anaerobic digestate application. *Environ. Pollut.* 268:115684. 10.1016/j.envpol.2020.115684 33010549 PMC7762785

[B42] Pokharel P. Qi L. Chang S. X. (2021). Manure-based biochar decreases heterotrophic respiration and increases gross nitrification rates in rhizosphere soil. *Soil Biol. Biochem.* 154:108147. 10.1016/j.soilbio.2021.108147

[B43] Prosser J. I. Nicol G. W. (2012). Archaeal and bacterial ammonia-oxidisers in soil: the quest for niche specialisation and differentiation. *Trends Microbiol.* 20 523–531. 10.1016/j.tim.2012.08.001 22959489

[B44] Sebilo M. Mayer B. Nicolardot B. Pinay G. Mariotti A. (2013). Long-term fate of nitrate fertilizer in agricultural soils. *Proc. Natl. Acad. Sci. U. S. A.* 110 18185–18189. 10.1073/pnas.1305372110 24145428 PMC3831475

[B45] Shen J. P. Zhang L. M. Zhu Y. G. Zhang J. B. He J. Z. (2008). Abundance and composition of ammonia-oxidizing bacteria and ammonia-oxidizing archaea communities of an alkaline sandy loam. *Environ. Microbiol.* 10 1601–1611. 10.1111/j.1462-2920.2008.01578.x 18336563

[B46] Shi R. Y. Ni N. Nkoh J. N. Li J. Y. Xu R. K. Qian W. (2019). Beneficial dual role of biochars in inhibiting soil acidification resulting from nitrification. *Chemosphere* 234 43–51. 10.1016/j.chemosphere.2019.06.030 31203040

[B47] Teutscherova N. Vazquez E. Masaguer A. Navas M. Scow K. M. Schmidt R. (2017). Comparison of lime- and biochar-mediated pH changes in nitrification and ammonia oxidizers in degraded acid soil. *Biol. Fertil. Soils* 53 811–821. 10.1007/s00374-017-1222-0

[B48] van Kessel M. A. Speth D. R. Albertsen M. Nielsen P. H. den Camp H. J. O. Kartal B. (2015). Complete nitrification by a single microorganism. *Nature* 528 555–559. 10.1038/nature16459 26610025 PMC4878690

[B49] Verhamme D. T. Prosser J. I. Nicol G. W. (2011). Ammonia concentration determines differential growth of ammonia-oxidising archaea and bacteria in soil microcosms. *ISME J.* 5 1067–1071. 10.1038/ismej.2010.191 21228892 PMC3131854

[B50] Wagner M. Loy A. Nogueira R. Purkhold U. Lee N. Daims H. (2002). Microbial community composition and function in wastewater treatment plants. *Antonie van Leeuwen. Inter. Gen. Molecu. Microbiol.* 81 665–680. 10.1023/A:1020586312170 12448762

[B51] Wang C. Zheng M. M. Song W. F. Wen S. L. Wang B. R. Zhu C. Q. (2017). Impact of 25 years of inorganic fertilization on diazotrophic abundance and community structure in an acidic soil in southern China. *Soil Biol. Biochem.* 113 240–249. 10.1016/j.soilbio.2017.06.019

[B52] Wang J. C. Wang J. L. Rhodes G. He J. Z. Ge Y. (2019a). Adaptive responses of comammox Nitrospira and canonical ammonia oxidizers to long-term fertilizations: implications for the relative contributions of different ammonia oxidizers to soil nitrogen cycling. *Sci. Total Environ.* 668 224–233. 10.1016/j.scitotenv.2019.02.427 30852199

[B53] Wang Z. Cao Y. Zhu-Barker X. Nicol G. W. Wright A. L. Jia Z. (2019b). Comammox Nitrospira clade B contributes to nitrification in soil. *Soil Biol. Biochem.* 135 392–395. 10.1016/j.soilbio.2019.06.004

[B54] Wang Z. Y. Zong H. Y. Zheng H. Liu G. C. Chen L. Xing B. S. (2015). Reduced nitrification and abundance of ammonia-oxidizing bacteria in acidic soil amended with biochar. *Chemosphere* 138 576–583. 10.1016/j.chemosphere.2015.06.084 26210022

[B55] Wertz S. Leigh A. K. Grayston S. J. (2011). Effects of long-term fertilization of forest soils on potential nitrification and on the abundance and community structure of ammonia oxidizers and nitrite oxidizers. *FEMS Microbiol. Ecol.* 79 142–154. 10.1111/j.1574-6941.2011.01204.x 22066501

[B56] Xiao Z. G. Rasmann S. Yue L. Lian F. Zou H. Wang Z. Y. (2019). The effect of biochar amendment on N-cycling genes in soils: a meta-analysis. *Sci. Total Environ.* 696:133984. 10.1016/j.scitotenv.2019.133984 31465924

[B57] Yu L. Homyak P. M. Kang X. X. Brookes P. C. Ye Y. K. Lin Y. N. (2019). Changes in abundance and composition of nitrifying communities in barley (*Hordeum vulgare* L.) rhizosphere and bulk soils over the growth period following combined biochar and urea amendment. *Biol. Fertil. Soils* 56 169–183. 10.1007/s00374-019-01410-6

[B58] Zhang X. Davidson E. A. Mauzerall D. L. Searchinger T. D. Dumas P. Shen Y. (2015). Managing nitrogen for sustainable development. *Nature* 528 51–59. 10.1038/nature15743 26595273

