# Supplementary material for: Ammonia- and Nitrite-Oxidizing Bacteria are Dominant in Nitrification of Maize Rhizosphere Soil Following Combined Application of Biochar and Chemical Fertilizer
Source: Front Microbiol. 2021 Oct 5;12:715070. doi: 10.3389/fmicb.2021.715070 (PMC8524134; doi:10.3389/fmicb.2021.715070)
Supplement: Supplementary file 1 [file Data_Sheet_1.docx]

*Supplementary Information*

**Ammonia-oxidizing bacteria and nitrite oxidizer are dominant in nitrification of maize rhizosphere soil following combined application of biochar and chemical fertilizer**

**Ping sun, Ziting Zhao, Pingshan Fan, Wei Chen, Yunze Ruan, Qing Wang***

Hainan Key Laboratory for Sustainable Utilization of Tropical Bio-resources, College of Tropical Crops, Hainan University, Haikou 570228, China

Corresponding author: Qing Wang Email:wangqing316000@163.com


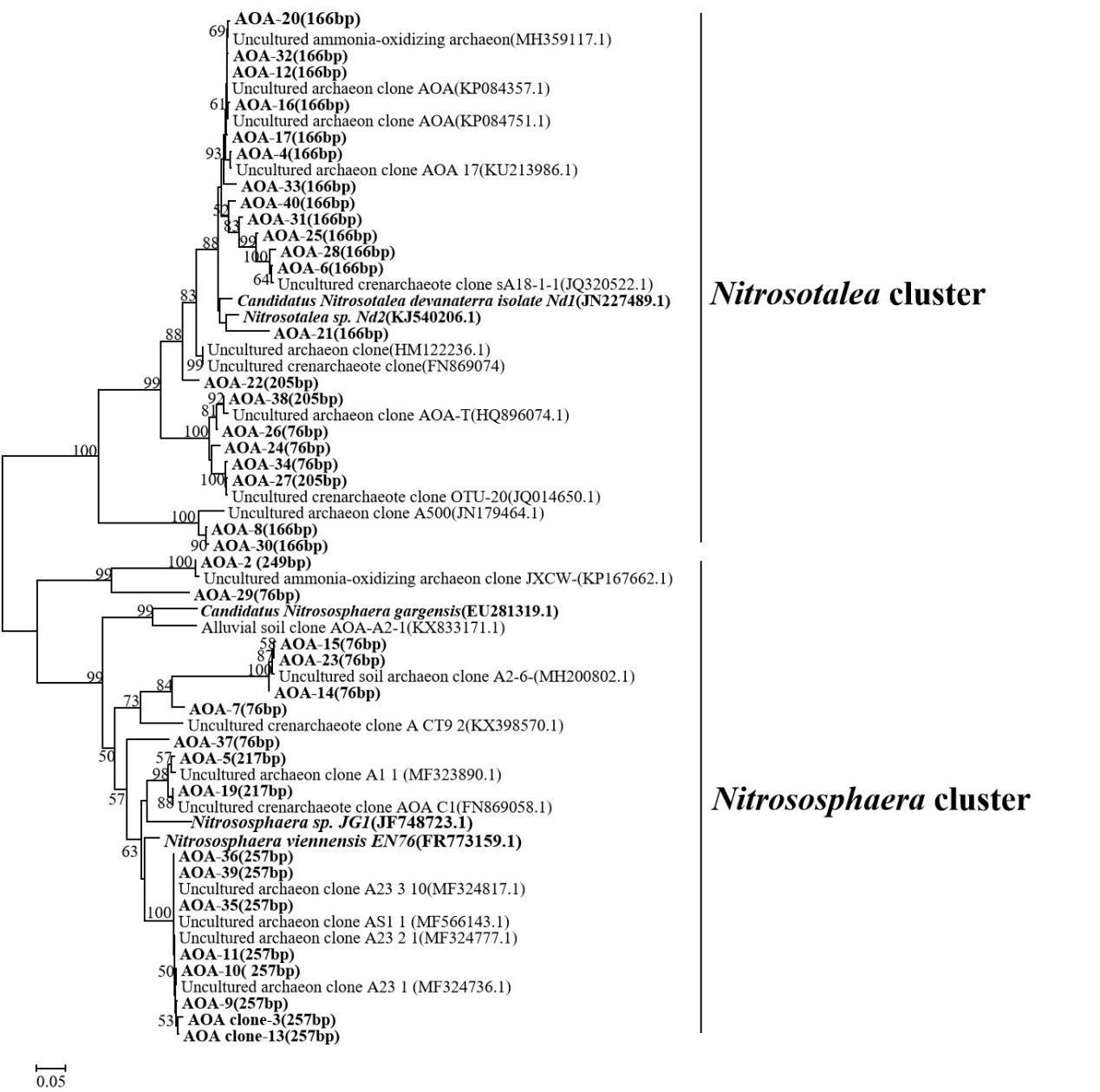


Figure S1 Neighbor-joining phylogenetic analysis of the representative sequences of ammonia-oxidizing archaea (AOA) *amoA* gene. The scale bar represents 5% nucleic acid sequence divergence, and bootstrap values (>50%) are indicated at branch points.


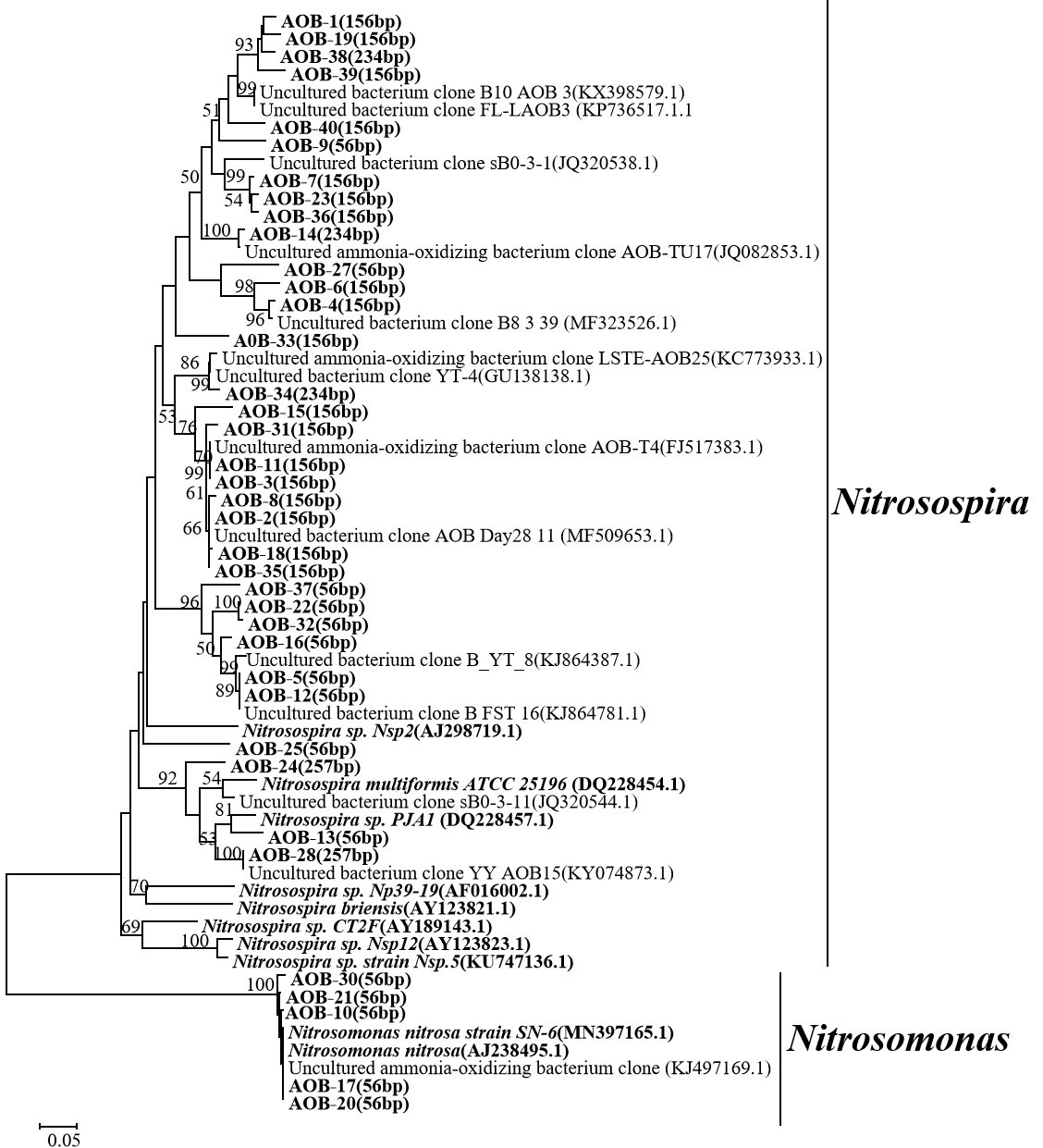


Figure S2 Neighbor-joining phylogenetic analysis of the representative sequences of ammonia-oxidizing bacteria (AOB) *amoA* gene. The scale bar represents 5% nucleic acid sequence divergence, and bootstrap values (>50%) are indicated at branch points.


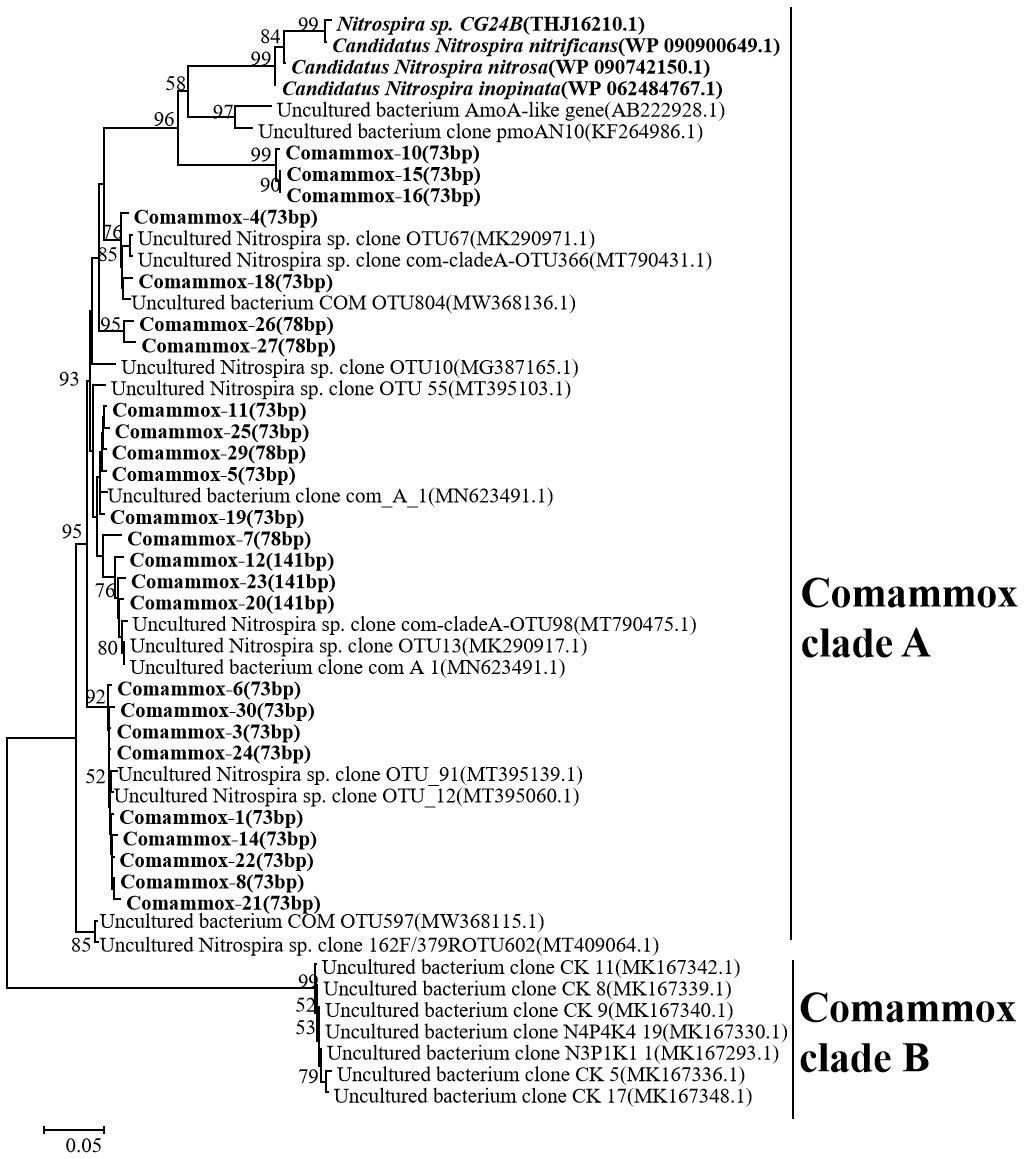


Figure S3 Neighbor-joining phylogenetic analysis of the representative sequences of Comammox *Nitrospira* (Comammox) *amoA* gene. The scale bar represents 5% nucleic acid sequence divergence, and bootstrap values (>50%) are indicated at branch points.


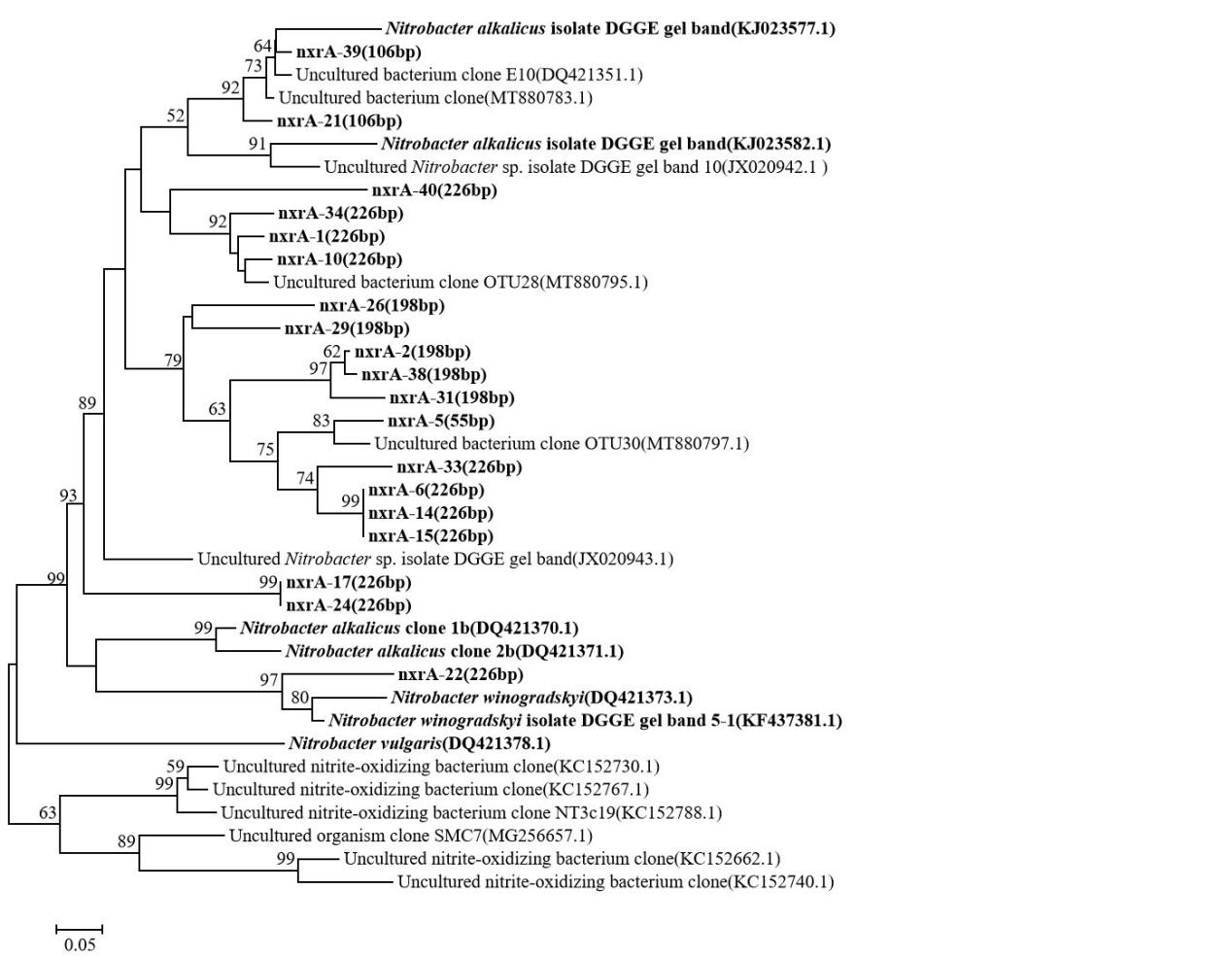


Figure S4 Neighbor-joining phylogenetic analysis of the representative sequences of *Nitrobacter* *nxrA* gene. The scale bar represents 5% nucleic acid sequence divergence,

and bootstrap values (>50%) are indicated at branch points.


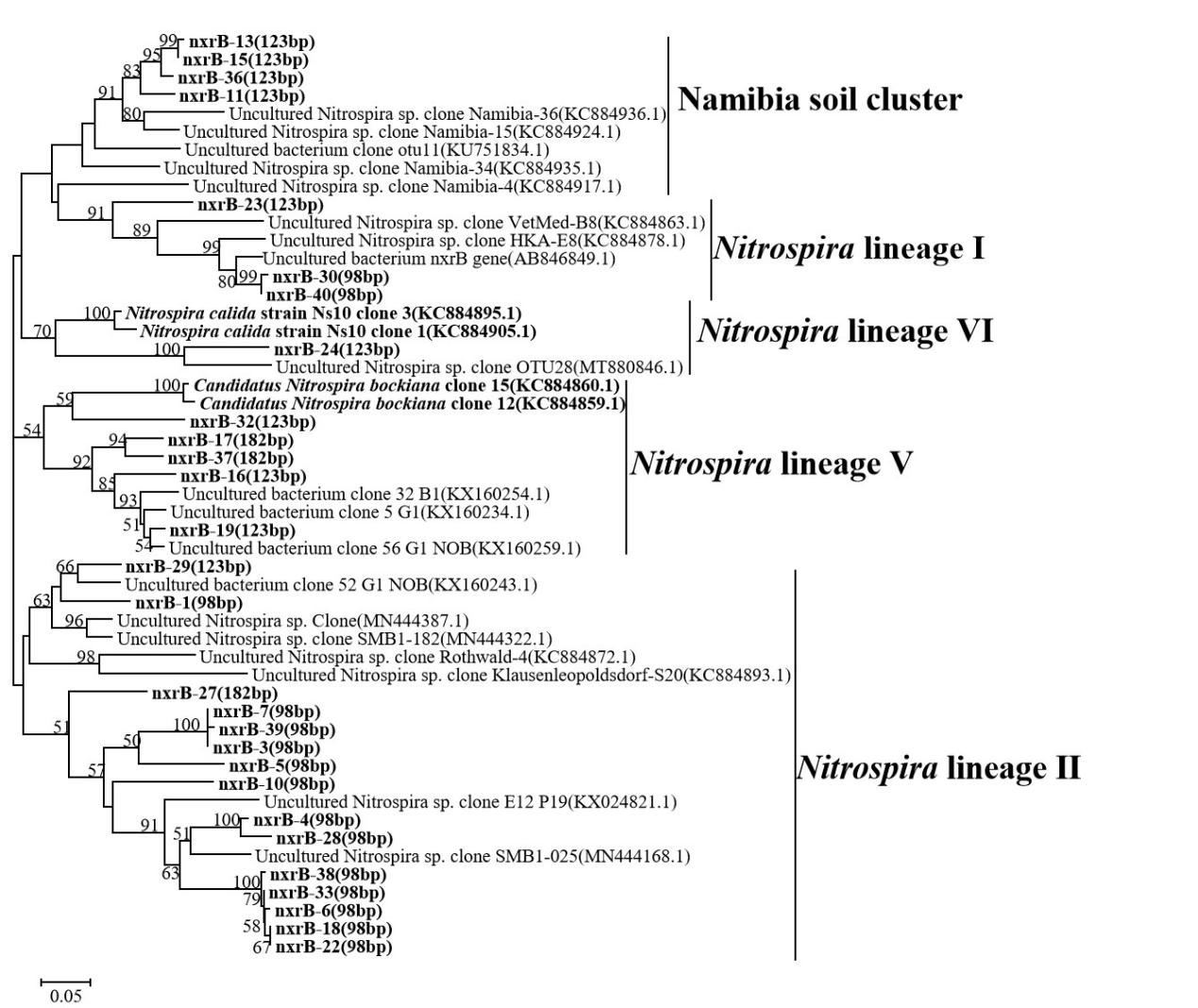


Figure S5 Neighbor-joining phylogenetic analysis of the representative sequences of *Nitrospira* *nxrB* gene. The scale bar represents 5% nucleic acid sequence divergence,

and bootstrap values (>50%) are indicated at branch points.
