# Supplementary material for: Ammonia- and Nitrite-Oxidizing Bacteria are Dominant in Nitrification of Maize Rhizosphere Soil Following Combined Application of Biochar and Chemical Fertilizer
Source: Front Microbiol. 2021 Oct 5;12:715070. doi: 10.3389/fmicb.2021.715070 (PMC8524134; doi:10.3389/fmicb.2021.715070)
Supplement: Supplementary file 2 [file Data_Sheet_2.docx]

*Supplementary Information*

**Ammonia-oxidizing bacteria and nitrite oxidizer are dominant in nitrification of maize rhizosphere soil following combined application of biochar and chemical fertilizer**

**Ping sun, Ziting Zhao, Pingshan Fan, Wei Chen, Yunze Ruan, Qing Wang***

Hainan Key Laboratory for Sustainable Utilization of Tropical Bio-resources, College of Tropical Crops, Hainan University, Haikou 570228, China

Corresponding author: Qing Wang Email:wangqing316000@163.com

**Table S1** Quantitative PCR primer sets and amplification conditions used in this study.

| **Target gene** | **Primer name** | **Primer sequence (5’-3’)** | **Quantitative PCR Amplification conditions** | **Length (bp)** | **Reference** |
| --- | --- | --- | --- | --- | --- |
| Comammox *Nitrospira* clade A *amoA* | comaA-244f_a | TACAACTGGGTGAACTA | 95°C for 10 min, 40 cycles of 94°C for  30 s, 54°C for 45 s and 72°C for 1 min, 83°C 10s, melting curve | 415 | Pjevac et al. (2017) |
|  | comaA-244f_b | TATAACTGGGTGAACTA |  |  |  |
|  | comaA-244f_c | TACAATTGGGTGAACTA |  |  |  |
|  | comaA-244f_d | TACAACTGGGTCAACTA |  |  |  |
|  | comaA-244f_e | TACAACTGGGTCAATTA |  |  |  |
|  | comaA-244f_f | TATAACTGGGTCAATTA |  |  |  |
|  | comaA-659r_a | AGATCATGGTGCTATG |  |  |  |
|  | comaA-659r_b | AAATCATGGTGCTATG |  |  |  |
|  | comaA-659r_c | AGATCATGGTGCTGTG |  |  |  |
|  | comaA-659r_d | AAATCATGGTGCTGTG |  |  |  |
|  | comaA-659r_e | AGATCATCGTGCTGTG |  |  |  |
|  | comaA-659r_f | AAATCATCGTGCTGTG |  |  |  |
| Comammox *Nitrospira* clade B *amoA* | comaB-244f_a | TAYTTCTGGACGTTCTA | 95°C for 10 min, 40 cycles of 94°C for  30 s, 54°C for 45 s and 72°C for 1 min, 83°C 10s, melting curve | 415 | Pjevac et al. (2017) |
|  | comaB-244f_b | TAYTTCTGGACATTCTA |  |  |  |
|  | comaB-244f_c | TACTTCTGGACTTTCTA |  |  |  |
|  | comaB-244f_d | TAYTTCTGGACGTTTTA |  |  |  |
|  | comaB-244f_e | TAYTTCTGGACATTTTA |  |  |  |
|  | comaB-244f_f | TACTTCTGGACCTTCTA |  |  |  |
|  | comaB-659r_a | ARATCCAGACGGTGTG |  |  |  |
|  | comaB-659r_b | ARATCCAAACGGTGTG |  |  |  |
|  | comaB-659r_c | ARATCCAGACAGTGTG |  |  |  |
|  | comaB-659r_d | ARATCCAAACAGTGTG |  |  |  |
|  | comaB-659r_e | AGATCCAGACTGTGTG |  |  |  |
|  | comaB-659r_f | AGATCCAAACAGTGTG |  |  |  |
| Archaeal *amoA* | CrenamoA23f | ATGGTCTGGCTWAGACG | 95°C for 10 min,40 cycles of 94°C for  30 s, 55°C for 30 s and 72°C for 1 min, 83°C 10s, melting curve | 635 | Tourna et al. (2008) |
|  | CrenamoA616r | GCCATCCATCTGTATGTCCA |  |  |  |
| Bacterial *amoA* | amoA-1F | GGGGTTTCTACTGGTGGT | 95°C for 10 min, 35 cycles of 94°C for  20 s, 56°C for 30 s and 72°C for 1 min, 83°C 10s, melting curve | 491 | Rotthauwe et al. (1997) |
|  | amoA-2R | CCCCTCKGSAAAGCCTTCTTC |  |  |  |
| Nitrobacter-like NOB | F1norA | CAGACCGACGTGTGCGAAAG | 95°C for 10 min, 40 cycles of 94°C for  20 s, 55°C for 30 s and 72°C for 1 min, 83°C 10s, melting curve | 322 | Han et al., (2018) |
|  | R2norA | TCCACAAGGAACGGAAGGTC |  |  |  |
| Nitrospira-like NOB | nxrB169f | TAC ATG TGG TGG AACA | 95°C for 10 min, 40 cycles of 94°C for  20 s, 56°C for 30 s and 72°C for 1 min, 83°C 10 s, melting curve | 480 | Han et al., (2018) |
|  | nxrB638r | CGG TTC TGG TCR ATCA |  |  |  |

**Table S2** PCR primer sets and amplification conditions for T-RFLP analysis used in this study.

| **Target gene** | **Primer name** | **Primer sequence (5’-3’)** | **PCR Amplification conditions** | **Restriction enzyme** | **Reference** |
| --- | --- | --- | --- | --- | --- |
| Archaeal *amoA* | CrenamoA23f | 6-FAM-ATGGTCTGGCTWAGACG | 95°C for 4 min, 35 cycles of 94°C for  30 s, 55°C for 45 s and 72°C for 1 min, 72°C for 8 min. | HpyCH4V | Wang et al., (2017) |
|  | CrenamoA616r | GCCATCCATCTGTATGTCCA |  |  |  |
| Bacterial *amoA* | amoA-1F | 6-FAM-GGGGTTTCTACTGGTGGT | 95°C for 4 min, 35 cycles of 94°C for  30 s, 56°C for 45 s and 72°C for 45 s, 72°C for 8 min. | MspI | Zhang et al., (2017) |
|  | amoA-2R | CCCCTCKGSAAAGCCTTCTTC |  |  |  |
| Comammox *Nitrospira* | Ntsp-amoA 162F | 6-FAM-GGATTTCTGGNTSGATTGGA | 95°C for 4 min, 35 cycles of 94°C for  30 s, 52°C for 30 s and 72°C for 40 s, 72°C for 6 min. | HhaI | Wang et al., (2019) |
|  | Ntsp-amoA 359R | WAGTTNGACCACCASTACCA |  |  |  |
| *Nitrobacter*-like NOB | F1norA | 6-FAM-CAGACCGACGTGTGCGAAAG | 95°C for 4 min, 35 cycles of 94°C for  30 s, 55°C for 45 s and 72°C for 40 s, 72°C for 6 min. | HaeIII | Ke et al., (2013) |
|  | R2norA | TCCACAAGGAACGGAAGGTC |  |  |  |
| *Nitrospira*-like NOB | nxrB169f | 6-FAM-TACATGTGGTGGAACA | 95°C for 4 min, 35 cycles of 94°C for  30 s, 56°C for 45 s and 72°C for 1 min, 72°C for 8 min. | HaeIII | Ke et al., (2013) |
|  | nxrB638r | CGG TTC TGG TCR ATCA |  |  |  |

**References**

Han, S., Zeng, L.Y., Luo, X.S., Xiong, X., Wen, S.L., Wang, B.R., et al. (2018). Shifts in *Nitrobacter*- and *Nitrospira*-like nitrite-oxidizing bacterial communities under long-term fertilization practices. Soil Biol. Biochem. 124, 118–125. doi: [10.1016/j.soilbio.2018.05.033](https://doi.org/10.1016/j.soilbio.2018.05.033)

Ke, X., Angel, R., Lu, Y., and Conrad, R. (2013). Niche differentiation of ammonia oxidizers and nitrite oxidizers in rice paddy soil. *Environ. Microbiol.* 15, 2275–2292. doi: 10.1111/1462-2920.12098

Pjevac, P., Schauberger, C., Poghosyan, L., Herbold, C. W., van Kessel, M. A. H. J., Daebeler, A., et al. (2017). AmoA-targeted polymerase chain reaction primers for the specific detection and quantification of comammox *Nitrospira* in the environment. Front. Microbiol. 8:1508. doi: 10.3389/fmicb.2017.01508

Rotthauwe, J.H., Witzel, K.P., and Liesack, W. (1997). The ammonia monooxygenase structural gene *amoA* as a functional marker: molecular fine-scale analysis of natural ammonia-oxidizing populations. *Appl. Environ. Microbiol.* 63, 4704–4712.[doi:10.1128/aem.63.12.4704-4712.1997](https://doi.org/10.1128/aem.63.12.4704-4712.1997)

Tourna, M., Freitag, T.E., Nicol, G.W., and Prosser, J.I. (2008). Growth, activity and temperature responses of ammonia-oxidizing archaea and bacteria in soil microcosms. *Environ. Microbiol.* 10,1357–1364. doi: 10.1111/j.1462-2920.2007.01563.x

Wang, J.C., Wang, J.L., Rhodes, G., He, J.Z., and Ge, Y. (2019a). Adaptive responses of comammox *Nitrospira* and canonical ammonia oxidizers to long-term fertilizations: implications for the relative contributions of different ammonia oxidizers to soil nitrogen cycling. *Sci. Total Environ.* 668, 224–233. doi: 10.1016/j.scitotenv.2019.02.427

Wang, Q., Liu, Y.R., Zhang, C.J., Zhang, L.M., Han, L.L., Shen, J.P., et al. (2017). Responses of soil nitrous oxide production and abundances and composition of associated microbial communities to nitrogen and water amendment. *Biol. Fertil. Soils* 53, 601-611. [doi:10.1007/s00374-017-1203-3](https://doi.org/10.1007/s00374-017-1203-3)

Zhang, C.J., Shen, J.P., Sun, Y.F., Wang, J.T., Zhang, L.M., Yang, Z.L., et al. (2017). Interactive effects of multiple climate change factors on ammonia oxidizers and denitrifiers in a temperate steppe. *FEMS Microbiol. Ecol.* 93(4). doi:10.1093/femsec/fix037
